# Neonatal Sepsis as Organ Dysfunction: Prognostic Accuracy and Clinical Utility of the nSOFA in the NICU—A Systematic Review

**DOI:** 10.3390/diagnostics16020349

**Published:** 2026-01-21

**Authors:** Bogdan Cerbu, Marioara Boia, Manuela Pantea, Teodora Ignat, Mirabela Dima, Ileana Enatescu, Bogdan Rotea, Andra Rotea, Vlad David, Daniela Iacob

**Affiliations:** 1 Doctoral School, Victor Babes University of Medicine and Pharmacy, 300041 Timisoara, Romania; bogdan.cerbu@umft.ro (B.C.); bogdan.rotea@umft.ro (B.R.); andra.alecu@umft.ro (A.R.); 2 Discipline of Neonatology, Victor Babes University of Medicine and Pharmacy, 300041 Timisoara, Romania; teodora.ignat@hosptm.ro (T.I.); dima.mirabela@umft.ro (M.D.); enatescu.ileana@umft.ro (I.E.); iacob.daniela@umft.ro (D.I.); 3 Department of Pediatric Surgery and Orthopedics, Victor Babes University of Medicine and Pharmacy, 300041 Timisoara, Romania; david.vlad@umft.ro

**Keywords:** sepsis, infant, newborn, intensive care units, neonatal, prognosis, severity of illness index

## Abstract

**Background and Objectives:** Early recognition of life-threatening organ dysfunction is central to modern sepsis frameworks. We systematically reviewed the prognostic performance and clinical utility of the Neonatal Sequential Organ Failure Assessment (nSOFA) for mortality and major morbidity in NICU populations. The search identified 939 records across databases; after screening and full-text assessment, 16 studies met the inclusion criteria. **Methods:** Following PRISMA guidance, we searched major databases (2019–2025) for observational or interventional studies reporting discrimination or risk stratification using nSOFA in neonates. Populations included suspected/proven infection and condition-specific cohorts. Heterogeneity in timing, thresholds, and outcomes precluded meta-analysis. **Results:** A cumulative sample exceeding 25,000 neonates was identified across late- and early-onset infection, all-NICU admissions, necrotizing enterocolitis, respiratory distress, and very preterm screening cohorts. Across settings and timepoints, nSOFA demonstrated consistent, good-to-excellent mortality discrimination, with reported AUROCs ≥ 0.80 and upper ranges near 0.90–0.92; serial scoring within the first 6–12 h generally improved risk classification. Disease-specific applications (NEC, early-onset infection) showed similar discrimination for death or composite adverse outcomes. **Conclusions:** Evidence from diverse NICU contexts indicates that nSOFA is a pragmatic, EHR-ready organ dysfunction score with robust discrimination for mortality and serious morbidity, supporting routine, serial use for risk stratification and standardized endpoints in neonatal sepsis pathways, aligned with contemporary organ dysfunction–based pediatric criteria.

## 1. Introduction

Neonatal sepsis remains a major driver of mortality and long-term morbidity in the NICU, especially among very preterm and very-low-birth-weight (VLBW) infants. Contemporary adult frameworks define sepsis as life-threatening organ dysfunction from dysregulated host response to infection and operationalize this with SOFA-based organ dysfunction scoring [1]. Recent global syntheses underscore the magnitude of the disease burden: pooled estimates suggest a neonatal sepsis incidence on the order of 2824 cases per 100,000 live births with an associated mortality/case-fatality of 17.6%, with disproportionate impact in preterm and very-low-birth-weight infants and marked regional variation. In parallel, Global Burden of Disease estimates have reported 1.3 million annual neonatal sepsis cases and 203,000 sepsis-attributable neonatal deaths worldwide [2,3]. At the population level, the global burden of sepsis remains substantial, with the highest incidence and mortality concentrated in low- and middle-income settings; neonates shoulder a disproportionate share of that burden [4,5]. Translating organ dysfunction constructs to neonates is therefore essential to bridge the historical gap between “infection” and “sepsis” in this population. The neonatal SOFA (nSOFA) score was proposed to quantify organ dysfunction with routine EHR variables (respiratory support/oxygenation, cardiovascular support, and platelet count) [1,4,5].

Pediatric critical care has already adapted SOFA principles: pSOFA demonstrated prognostic validity in children [2], and the 2024 Phoenix consensus provided modern pediatric sepsis/shock criteria anchored in organ dysfunction [3]. Yet neonates—particularly preterm infants—differ physiologically, and consensus neonatal definitions remain unsettled, underscoring the need for neonatal-specific tools [6]. In this context, nSOFA offers a developmentally sensitive, EHR-friendly approach that aligns neonatal sepsis phenotyping with broader sepsis science while remaining feasible for point-of-care and research use. This convergence (pSOFA/Phoenix for older children, nSOFA for neonates) creates an opportunity to harmonize sepsis definitions across the pediatric age span, support antibiotic stewardship, and refine eligibility/enrichment in neonatal trials [2,3,6]. Importantly, organ dysfunction scoring does not replace infection ascertainment. In neonates—particularly very preterm infants—respiratory and hemodynamic deterioration may be infectious or noninfectious, and sepsis evaluation remains anchored in cultures, clinical contexts, and adjunct diagnostics. Biomarkers (C-reactive protein, procalcitonin, and emerging markers such as presepsin) may support infection-likelihood assessment, while nSOFA primarily quantifies the severity and trajectory of organ dysfunction once clinical concern is present [6,7,8]. This distinction is central when interpreting risk stratification results and when considering implementation into sepsis pathways.

The nSOFA domains aim to assess infection-driven neonatal organ dysfunction: escalating ventilatory/oxygen needs, vasopressor/corticosteroid support for hemodynamic compromise, and thrombocytopenia reflecting dysregulated inflammation/coagulation. In the original VLBW, late-onset sepsis (LOS), higher nSOFA values at evaluation and at +6 h/+12 h strongly discriminated mortality risk [7]. Subsequent multicenter work in preterm infants with suspected/proven late-onset infection (LOI) replicated and extended these findings, showing improved discrimination at +6 h and +12 h versus the initial evaluation [8]. Beyond infection episodes, nSOFA has also been validated for all-cause NICU mortality risk across gestational ages and birth-weight, supporting its general prognostic utility [9].

Several cohorts demonstrate that serial scoring after sepsis evaluation enhances discrimination versus a single baseline measurement, with +6 h/+12 h often outperforming time-0 [8,9]. This pattern appears in different clinical contexts—suspected LONS where +6 h had the strongest AUC for mortality and severe morbidity [10,11], and in high-resolution trajectory studies of extremely preterm infants where cumulative and hourly nSOFA dynamics linked to adverse outcomes [12]. Related conditions such as necrotizing enterocolitis (NEC), which shares inflammatory and hemodynamic pathways with infection-associated organ injury, also show nSOFA associations with mortality, suggesting pathobiologic coherence beyond bacteremia alone [10]. Early-life applications reveal that nSOFA can anticipate important respiratory and composite morbidities, potentially extending its role from acute sepsis phenotyping to broader risk prognostication in fragile preterms [13]. Head-to-head comparisons further indicate nSOFA’s superiority to SIRS criteria for predicting LONS mortality and highlight the value of repeated measures [14].

For a consensus LOI/LONS sepsis definition, clinicians need both an organ dysfunction threshold and a time anchor. nSOFA provides both: it can separate “infected only” from “septic” infants at evaluation and refine risk within the first 6–12 h as physiology evolves—windows that align with antibiotic, respiratory, and hemodynamic decision-making. Its use as a consistent, EHR-captured endpoint could also enrich trials and harmonize outcome reporting across NICUs. Moreover, beyond mortality, nSOFA has shown utility in anticipating short-term respiratory outcomes and informing multi-morbidity risk, complementing infection diagnostics and potentially curbing unnecessary escalation in low-risk infants [9,11,15]. From an implementation perspective, nSOFA can support standardized escalation triggers, repeated bedside reassessment, and comparability of outcomes across studies and quality-improvement initiatives. Its reliance on routinely captured variables makes it well-suited for automated EHR calculation and trend-based alerts in real-time NICU workflows.

We hypothesize that the nSOFA score demonstrates strong discrimination for mortality in neonatal sepsis and related inflammatory conditions, and that serial assessments outperform time-zero scoring for predicting death and serious morbidity. We further hypothesize that low thresholds identify infants at meaningful risk and that nSOFA compares favorably with commonly used neonatal severity models and outperforms non-organ dysfunction criteria for late-onset sepsis. The primary objective of this review is to synthesize study-level evidence on (i) nSOFA’s mortality discrimination across clinical contexts, (ii) timing windows and thresholds that optimize performance, and (iii) associations with non-mortality outcomes.

## 2. Materials and Methods

### 2.1. Protocol and Registration

Following PRISMA guidance [16], we searched PubMed/MEDLINE, Scopus, and Web of Science (2019–2025) for studies evaluating nSOFA in neonates and reporting prognostic performance for mortality and/or major morbidity; 16 studies met the inclusion criteria (Supplementary File S1). Our objective was to synthesize the open-access evidence on nSOFA in neonatal sepsis and critical illness, focusing on (i) discrimination for mortality, (ii) thresholds and time-window performance, and (iii) associations with non-mortality morbidities (BPD, ROP, ventilation). Because several papers span different phenotypes, we prespecified inclusion if nSOFA was calculated in neonates and quantitatively linked to mortality and morbidity.

We did not restrict by gestational age, sepsis definition (suspected vs. proven), or center type (single vs. multicenter). Given clear heterogeneity in populations, time windows (T−6 to T+48 h around sepsis evaluation; first 72 h after birth), and outcome definitions, meta-analysis was not attempted. The protocol emphasized transparent handling of missingness (NR when data were not reported), explicit study-level sourcing for every table datum, and careful distinction between mortality vs. morbidity outcomes. This review did not require IRB approval. We registered the protocol in the Open Science Framework (OSF). Ethics committee approval was not required because this study is a systematic review of previously published, de-identified data and involved no direct interaction with human participants.

The PICO statement was considered as follows: Population (P): Neonates in NICU or special-care nurseries undergoing evaluation for infection or with related pathologies where organ dysfunction is pertinent. Index (I): nSOFA score (0–15) computed at evaluation and/or serially, either manual or EHR-derived. Comparator (C): No score/clinical judgment, earlier time points, alternative thresholds, or other scores when provided. Outcomes (O): Primary—mortality (episode-specific or in-hospital); Secondary—serious morbidity, composite adverse outcomes, respiratory endpoints, and operating characteristics.

### 2.2. Eligibility Criteria

Inclusion criteria were as follows: (1) Population: Neonates cared for in an NICU or special-care nursery; any gestational age, with emphasis on preterm/VLBW cohorts. (2) Index measure: nSOFA score (0–15) calculated from respiratory, cardiovascular, and hematologic components—manual or EHR-derived. (3) Outcomes: Mortality (all-cause or sepsis-related), and/or predefined neonatal morbidities (BPD, ROP, surgical NEC, ventilation endpoints). (4) Design: Observational cohorts (single or multicenter), retrospective or prospective. (5) Availability: Open-access full text on PubMed, Scopus, and Web of Science. Exclusion: Studies not using nSOFA; commentary/editorials without original data; adult/pediatric (non-neonatal) SOFA; paywalled reports without accessible data; duplicate analyses of the same cohort without new outcomes.

We accepted studies outside classic sepsis phenotypes when the index measure remained nSOFA and outcomes were clinically important, because neonatal organ dysfunction pathways overlap (septic shock, hypoxic respiratory failure), and such contexts inform external validity and broaden use cases. We did not enforce a minimum sample size, as several nSOFA investigations are single-center and time-dense, yet highly informative regarding thresholds and temporal windows.

### 2.3. Information Sources and Search Strategy

PubMed (last searched 22 September 2025; filters: Humans, Newborn; English; Free full text when possible): (“neonatal sequential organ failure” OR nSOFA[tiab] OR “neonatal SOFA”[tiab]) AND (sepsis[tiab] OR infection[tiab] OR “late-onset”[tiab] OR “early-onset”[tiab] OR NEC[tiab] OR “respiratory distress syndrome”[tiab] OR RDS[tiab]) AND (mortality[tiab] OR death[tiab] OR morbidity[tiab] OR BPD[tiab] OR ROP[tiab] OR surgery[tiab] OR outcome*[tiab] OR AUROC[tiab]) with complementary MeSH expansion: (“Sepsis”[Mesh] OR “Infection”[Mesh]) AND (“Infant, Newborn”[Mesh]) AND (“Organ Dysfunction Scores”[Mesh] OR SOFA[tiab] OR nSOFA[tiab]).

Scopus (TITLE-ABS-KEY): TITLE-ABS-KEY((“neonatal sequential organ failure” OR nSOFA OR “neonatal SOFA”) AND (sepsis OR “late-onset” OR “early-onset” OR infection OR NEC OR “respiratory distress syndrome” OR RDS) AND (mortality OR death OR morbidity OR BPD OR ROP OR surgery OR outcome* OR AUROC)) with document type = Article; language = English.

Web of Science Core Collection (Topic search = title/abstract/keywords): TS = (“neonatal sequential organ failure” OR nSOFA OR “neonatal SOFA”) AND TS = (sepsis OR infection OR “late-onset” OR “early-onset” OR NEC OR “respiratory distress syndrome” OR RDS) AND TS = (mortality OR death OR morbidity OR BPD OR ROP OR surgery OR outcome* OR AUROC); refined by Research Areas = Pediatrics; document types = Article.

**Figure 1:**
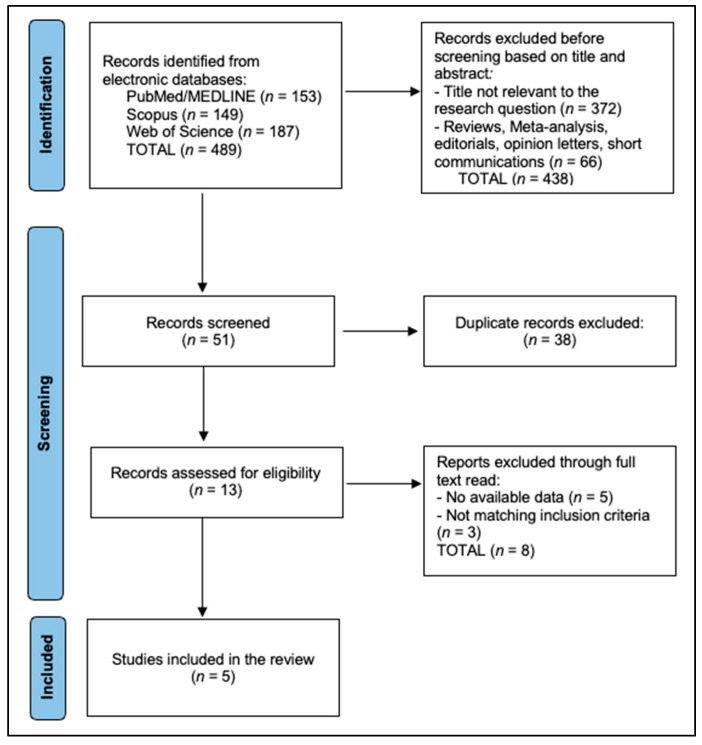
PRISMA Flowchart Diagram.

The PRISMA flow diagram shows that 939 records were identified across databases (PubMed/MEDLINE = 316, Scopus = 241, Web of Science = 382). Following title/abstract screening, 873 records were excluded—812 as not relevant and 61 due to ineligible article types (reviews, meta-analyses, editorials, opinion letters, short communications)—leaving 66 records for screening. Deduplication removed 43, resulting in 23 reports assessed at full text. In total, 7 reports were then excluded (4 with no available data and 3 not meeting inclusion criteria), yielding 16 studies included in the review, as seen in Figure 1.

### 2.4. Study Selection and Data Extraction

Two reviewers screened the studies and included every unique study that (i) used nSOFA in neonates and (ii) reported at least one quantitative association with mortality or morbidity. We extracted the first author/year, country/setting, design (single vs. multicenter), population (very preterm, VLBW, RDS), number of infants/episodes, sepsis phenotype (EOS/LOS/suspected LOS), time windows (T0, +6 h, +12 h; −6 to +48 h), mortality metrics (AUROC/AUC at specified windows, OR/HR per 1-point increment, threshold-based odds), and non-mortality outcomes (BPD, ROP, ventilation day-5, NEC surgery). If a value could not be found in the open text/abstract, it is marked NR. Disagreements in extraction were resolved by consensus. All data were extracted at the study level from eligible published reports identified through database searching (PubMed/MEDLINE, Scopus, Web of Science); no individual-level patient data were accessed.

Meta-analysis is desirable when studies are sufficiently comparable to support pooled effect estimates (common timing anchors, thresholds, outcomes, and reporting of uncertainty). In the nSOFA literature [17], however, included studies differed substantially in phenotype (suspected vs. culture-proven infection; EOS vs. LOS; NEC/RDS/all-NICU cohorts), timing windows (evaluation, +6/+12/+24 h, peak within 72 h, or dense trajectories), and outcome definitions (episode-specific mortality, in-hospital mortality, composite morbidity endpoints). In addition, several studies did not report the variance measures required for quantitative pooling. Therefore, we prespecified and performed a structured qualitative synthesis with standardized extraction and descriptive aggregation rather than a pooled meta-analysis.

### 2.5. Risk of Bias

We qualitatively appraised selection, measurement, and reporting biases. Typical concerns include retrospective design, single-center sampling, variable sepsis definitions (suspected vs. culture-proven), heterogeneous windows (evaluation vs. +6/+12 h), and differing covariate adjustments. Multicenter EHR studies mitigate center-specific biases but may include mixed illness acuity beyond sepsis. Thresholds (≥2, ≥4) were not uniform; cut-point optimization risks overfitting in small cohorts.

Where available, we present study-reported AUROC values and adjusted effect estimates (OR/HR) in tabular form and in a figure for visual comparison. Because timing anchors, thresholds, and outcomes were not sufficiently uniform—and variance measures were inconsistently reported—we did not compute pooled effect estimates.

## 3. Results

Table 1 summarizes the 16 included studies [18,19,20,21,22,23,24,25,26,27,28,29,30,31,32,33] and shows that research on the Neonatal Sequential Organ Failure Assessment score has been conducted across multiple countries, designs, and neonatal intensive care unit populations, with sample sizes ranging from small single-center cohorts (for example 60 infants in a bacteremic late-onset sepsis cohort [24] and 104 infants in an early-onset infection cohort [28]) to very large multicenter datasets (20,152 neonatal intensive care unit admissions in a validation study [23]). Most studies focused on preterm infants evaluated for late-onset infection or late-onset neonatal sepsis (for example 259 infants [18], 706 infants [21], 1574 infants [29], and 1481 evaluations [26]), but the table also shows broader “beyond sepsis” applications, including necrotizing enterocolitis (259 infants [32]) and respiratory distress syndrome (1281 infants [20]), as well as early postnatal prediction work within the first 72 h after birth in very preterm infants (238 infants [19] and 423 infants [31]). The timing of assessment varied widely, but most studies used an “at evaluation” score and then repeated scoring at 6 and/or 12 h (for example [18,21,24,25,30]), while others used admission values across the whole neonatal intensive care unit stay [20,23,33] or dense-time-course approaches around the evaluation window [27]. Overall, the table highlights that the evidence comes from heterogeneous but clinically relevant settings, supporting broad external validity while also explaining why pooled meta-analysis is difficult.

Table 2 reports how well the Neonatal Sequential Organ Failure Assessment score separates infants who die from those who survive, mainly using the area under the receiver operating characteristic curve, and it shows a consistent pattern: performance is often good at the time of evaluation and becomes better when the score is repeated 6–12 h later. In the original very-low-birth-weight late-onset sepsis cohort, the area under the receiver operating characteristic curve increased from 0.77 at evaluation to 0.93 at 12 h [24], and a score of four or more at 12 h separated mortality risk strongly (71% vs. 7%) [24]. In a multicenter, preterm late-onset infection study, the best-performing time window reached 0.88 overall (with center-level ranges up to the mid-0.90s), and performance generally improved at 6 h compared with evaluation [18]. One study directly compared this organ dysfunction score with systemic inflammatory response syndrome criteria and found much higher discrimination at evaluation (0.95 vs. 0.569) with an optimal evaluation cut-point of four [25]. In a large cohort of all late-onset infection evaluations, repeating the score at 6 h improved the area under the receiver operating characteristic curve from 0.76 to 0.82, and a low cut-point of two or more achieved high sensitivity (87%) with a very high negative predictive value (99%), meaning death was very unlikely when the score stayed below that threshold in that dataset [26]. Strong discrimination was also reported in other contexts, including a 12 h value of 0.91 in very-low-birth-weight infants [22], 0.92 in a Brazilian very-low-birth-weight late-onset neonatal sepsis cohort [29], and good discrimination for death (0.87) and for surgery-or-death (0.84) in necrotizing enterocolitis [32]. A comparison study in preterm infants at admission found the Neonatal Sequential Organ Failure Assessment score outperformed two established neonatal risk scores for mortality prediction (0.90 vs. 0.82 and 0.79) [33]. Taken together, the table supports a simple clinical message for non-specialists: repeating the score a few hours after the first assessment often provides clearer separation of high-risk and low-risk infants than relying on a single initial value.

**Figure 2:**
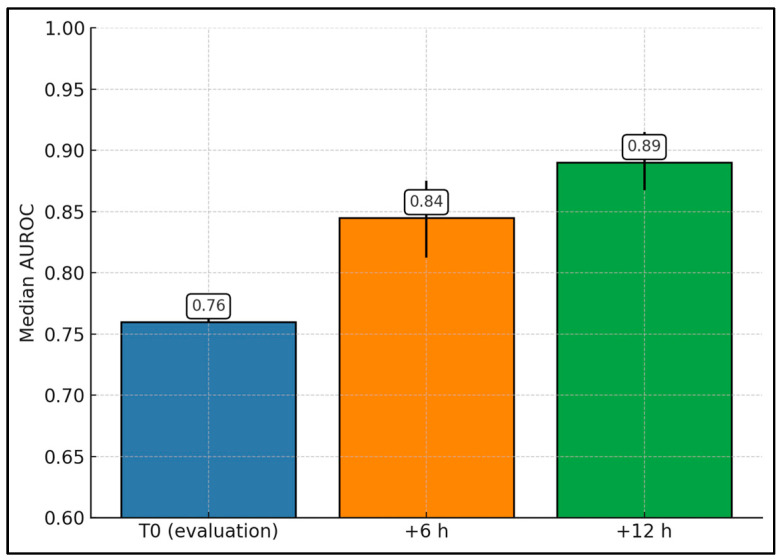
nSOFA mortality discrimination by time window (aggregate across studies).

Figure 2 aggregates AUROCs for mortality at T0, +6 h, and +12 h from multiple cohorts. The median AUROC increased from 0.76 at T0 to 0.85 at +6 h (IQR ≈ 0.81–0.88) and 0.89 at +12 h (IQR ≈ 0.87–0.92), supporting the practice-relevant concept that serial scoring outperforms baseline. Collectively, these descriptive aggregates support a consistent direction of effect—a higher nSOFA, particularly when reassessed at +6 to +12 h, corresponds to higher mortality and adverse outcome risk—and provide a practical quantitative summary in the setting of heterogeneity that precludes statistical pooling.

Table 3 translates score performance into clinical outcomes by showing what increases in the Neonatal Sequential Organ Failure Assessment score mean for mortality and major complications, and it emphasizes that higher scores generally correspond to substantially higher risk across multiple neonatal intensive care unit conditions. Several studies did not report adjusted effect sizes, but still showed clinically large risk separation at practical cut-points (for example, a score of four or more strongly separated mortality at evaluation, 6 h, and especially 12 h in very-low-birth-weight late-onset sepsis [24], and higher scores were linked to markedly higher crude and adjusted mortality odds across multiple time windows in very preterm late-onset sepsis, often cited as approximately 7–16 times higher crude odds and approximately 9–18 times higher adjusted odds when scores were above four [27]). Where adjusted estimates were reported, the direction and size of effect were consistent: in suspected late-onset sepsis among preterm infants, each one-point increase at 6 h was associated with higher 10-day mortality (adjusted odds ratio 1.31, 95% confidence interval 1.22–1.40) and also higher odds of bronchopulmonary dysplasia and retinopathy of prematurity (adjusted odds ratios 1.30 and 1.24, respectively) [21]. In respiratory distress syndrome, mortality rose with each one-point increase (adjusted hazard ratio 1.48, 95% confidence interval 1.32–1.67), and infants in a higher-score group had far greater mortality risk than those in a lower-score group (adjusted hazard ratio 19.35, 95% confidence interval 4.41–84.95) [20]. For respiratory outcomes after late-onset sepsis in preterm infants, higher scores were linked to escalation in respiratory support by 24 h and to invasive ventilation (odds ratios 1.68 and 1.56 per point), as well as failure to return to baseline respiratory support (odds ratio 1.39 per point), and higher scores also correlated with severe retinopathy of prematurity (odds ratio 1.30) [30]. Importantly for non-specialists, this table supports an intuitive interpretation: when the score increases over the first hours of illness, the likelihood of death and serious complications increases meaningfully, and serial reassessment is clinically informative across sepsis, necrotizing enterocolitis [32], respiratory distress syndrome [20], and broad neonatal intensive care unit admission cohorts [23].

**Figure 3:**
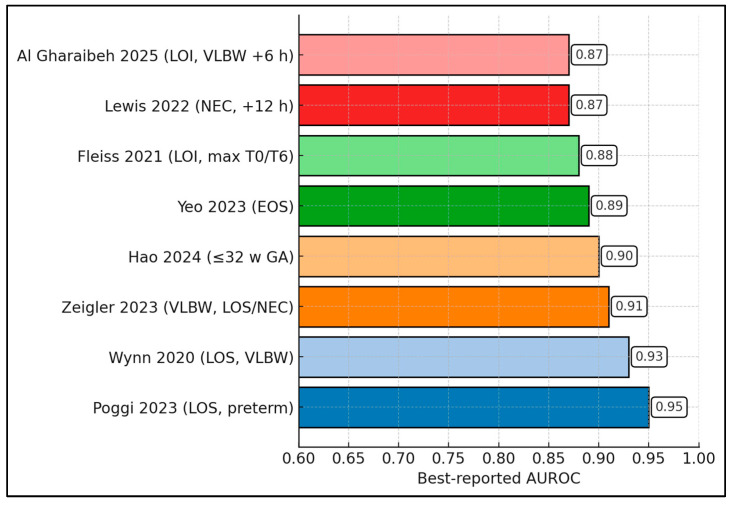
Best AUROC per study [18,22,24,25,26,28,32,33].

Across eight cohorts, the best-reported nSOFA AUROC per study is consistently high (range 0.87–0.95; median 0.90; IQR 0.875–0.92). The highest value appears in Poggi et al. (0.95) [25], followed by Wynn et al. (0.93) [24] and Zeigler et al. (0.91) [22]. In very preterm populations, Hao atl al. reports 0.90 [33] and Yeo et al. 0.89 [28]. Multicenter LOI work shows Fleiss et al. at 0.88 [18], while NEC-focused analysis yields Lewis et al. 0.87 [32]; Al Gharaibeh et al. also peaks at 0.87 in LOI (including VLBW at +6 h) [26]. Because each value reflects that study’s optimal time window (commonly +6 h or +12 h), the figure demonstrates that, across LOS/LOI, EOS, and NEC contexts, nSOFA achieves excellent discrimination (≥0.85) when measured at its best-performing time point (Figure 3).

## 4. Discussion

### 4.1. Summary of Evidence

The temporal improvement in nSOFA discrimination from evaluation to +6 and +12 h mirrors a broader move from static, admission-only neonatal risk indices to dynamic, physiology-tracking frameworks. Foundational admission scores such as CRIB-II and SNAPPE-II achieved excellent one-time mortality discrimination in derivation/validation cohorts but were not designed for frequent recalculation around evolving infection physiology or treatment response [34,35]. By contrast, serial nSOFA captures early cardiorespiratory and hematologic derangements that meaningfully re-stratify risk within hours—precisely during antibiotic, ventilatory, and vasoactive decision windows—aligning neonatal practice with organ dysfunction-anchored approaches used in older children and adults. These features help explain the consistently higher AUCs observed at +6 to +12 h compared with time-zero across heterogeneous cohorts.

Compared with earlier multi-organ dysfunction constructs developed for the NICU, nSOFA offers a leaner, EHR-friendly footprint without sacrificing conceptual fidelity to neonatal organ failure. NEOMOD and modified NEOMOD track additional systems and require broader laboratory inputs, enhancing physiologic completeness but limiting recalculation frequency and cross-unit portability [36,37]. Focusing on three domains—respiratory support/oxygenation, vasoactive or steroid use, and platelet count—nSOFA preserves the core pathophysiology while enabling granular, repeated assessments; the observed step-up in performance at +6 to +12 h likely reflects this balance between parsimony and responsiveness.

Applicability across clinical contexts—including late-onset/sepsis evaluations, early-onset infection, and NEC—supports nSOFA as a transportable organ dysfunction framework rather than a bacteremia-specific tool. Recent work in term neonates further demonstrates that both pSOFA and nSOFA predict mortality with good to very good discrimination across NICU and PICU/PCICU settings, underscoring the value of harmonized, age-calibrated organ dysfunction scoring across the pediatric continuum [38]. Against this backdrop, the present synthesis—showing stronger discrimination with early serial reassessment—suggests that nSOFA adds prognostic clarity beyond admission-only indices and can support trial enrichment and standardized outcome reporting irrespective of culture status or sepsis phenotype.

At the physiology level, the prominence of respiratory and cardiovascular components is consistent with evidence that cardio-respiratory instability often precedes overt sepsis. Randomized implementation of heart-rate-characteristic monitoring in very-low-birth-weight infants reduced mortality, indicating that physiology-first signals can influence outcomes when coupled to timely action [39]. Such physiomarkers and repeated nSOFA may be complementary: early alerts can flag occult instability, while nSOFA quantifies downstream organ impact that tracks near-term death and severe morbidity. The observed gains in discrimination at +6 to +12 h are therefore biologically and operationally coherent with a “physiology-to-organ dysfunction” cascade.

Interpretation of the hematologic domain benefits from contextualization with transfusion trials. Thrombocytopenia frequently accompanies neonatal sepsis and rightly increases the hematologic nSOFA subscore, yet the PlaNeT-2/MATISSE randomized trial demonstrated higher rates of death or major bleeding with a liberal prophylactic platelet threshold (50 × 10^9^/L) compared with a restrictive threshold (25 × 10^9^/L); subsequent analyses showed the advantage of low thresholds across baseline-risk strata [40,41]. These findings suggest that elevated nSOFA due to thrombocytopenia should prompt intensified evaluation and organ-support optimization, while transfusion decisions adhere to evidence-based restrictive strategies rather than attempts to normalize platelet counts prophylactically.

Nevertheless, definitional heterogeneity remains a barrier to synthesis and trial design in neonatal sepsis. A systematic review of randomized trials cataloged wide variation in sepsis definitions that, unlike adult/pediatric consensus, often did not anchor on organ dysfunction [42]. In this context, the combination of time-anchored thresholds (evaluation and +6 to +12 h) and demonstrated feasibility of EHR automation for real-time nSOFA computation provides a pragmatic path toward harmonized enrollment criteria and outcomes across centers [43]. The pattern observed here—good discrimination at evaluation and consistently stronger discrimination with early serial scoring—offers practical parameters for consensus and for prospective interventional studies.

Because respiratory and cardiovascular instability are common in preterm neonates and may reflect sepsis or noninfectious decompensation, adjunct biomarkers can support clinical probability assessment alongside organ dysfunction scoring [44]. Procalcitonin and presepsin have been investigated as markers that may improve early discrimination of bacterial infection in pediatric and neonatal contexts, and may be particularly useful when integrated with serial physiologic assessment. However, biomarker performance varies by gestational age, postnatal age, and comorbidity, and presepsin is not universally available across NICUs. In this framework, nSOFA is best interpreted as a pragmatic, EHR-ready organ dysfunction severity instrument that can be paired with infection-focused diagnostics (cultures, clinical evaluation, and biomarkers) to improve sepsis phenotyping and risk stratification.

Embedding nSOFA into NICU sepsis pathways can operationalize organ dysfunction-based triage, particularly when scored at evaluation and repeated within 6–12 h. In aggregate, cohorts > 25,000 infants show nSOFA achieves good-to-excellent discrimination (typically AUROC ≈ 0.8–0.9), enabling early identification of high-risk infants for escalation bundles (respiratory/circulatory support) while providing reassurance for low-risk trajectories. Integration with electronic health records facilitates automated alerts and trend monitoring, harmonizing neonatal practice with pediatric organ dysfunction criteria and Sepsis-3 concepts.

### 4.2. Limitations

The evidence base is dominated by retrospective cohorts from higher-resource centers, with heterogeneity in case definitions, scoring intervals, and thresholds. Many reports emphasize discrimination while providing limited calibration or decision-analytic metrics; external validity to term neonates and lower-resource settings remains incompletely characterized. Variation in supportive-care practices (e.g., ventilation and transfusion policies) may influence component subscores and transportability. Prospective impact-of-implementation studies and head-to-head comparisons with legacy illness-severity indices are fewer than ideal.

## 5. Conclusions

Across diverse NICU populations and disease contexts, nSOFA consistently demonstrates good-to-excellent prognostic discrimination for mortality and clinically important morbidity, with performance typically improving when scores are reassessed within 6–12 h after initial evaluation. The literature supports nSOFA as a pragmatic, EHR-ready organ dysfunction score for serial risk stratification and standardized outcome reporting; however, variability in timing anchors, thresholds, and limited calibration reporting highlight priorities for prospective multicenter implementation studies and harmonized reporting standards.

## Figures and Tables

**Table 1:** Study characteristics.

First Author (Year)	Country/Region	Setting/Design	Population/Phenotype	N (Infants/Episodes)	Time Window/Context
Fleiss (2021) [18]	US multicenter	Multicenter cohort	Preterm infants with late-onset infection	259	Evaluation; serial (+6, +12 h)
Xu (2023) [19]	China	Single-center, retrospective	Very preterm (≤32 wk) within 72 h after birth; BPD outcome	238	Early postnatal (≤72 h)
Shi (2022) [20]	China	Retrospective cohort	Neonates with RDS in NICU	1281	Admission; whole stay
Kurul (2024) [21]	Netherlands	Preterm with suspected LOI	Morbidity and mortality	706	T0, +6, +12 h (serial)
Zeigler (2023) [22]	US	VLBW; HeRO vs. nSOFA	Sepsis/mortality prediction	956	Evaluation; +12 h
Wynn (2021) [23]	US multicenter	Validation study	All NICU admissions (prognosis)	20,152	Admission and serial windows
Wynn (2020) [24]	US single-center	Bacteremic VLBW with LOS	Mortality discrimination	60	T0, +6, +12 h
Poggi (2023) [25]	Italy	Retrospective	≤32 wk with LONS	112	T0, +6, +12, +24 h; vs. SIRS
Al Gharaibeh (2025) [26]	US	Single-center, retrospective	All neonates with LOI evaluations	1481	T0 and +6 h; eval-specific mortality
Kraja (2024) [27]	Turkey	Single-center, retrospective	Very preterm with culture-proven LOS	106	9 time points incl. pre-/post-eval up to 48 h
Yeo (2023) [28]	Multicenter	Prospective cohort	Early-onset infection	104	Evaluation; serial; mortality
Lobo (2022) [29]	Brazil	Retrospective	VLBW with LONS	1574	Evaluation; mortality predictor
Poggi (2025) [30]	Italy	Retrospective	Preterm with LONS; respiratory outcomes	NR	T0/+6/+12/+24; respiratory endpoints
Berka (2022) [31]	Czech Republic	Single-center	<32 wk within 72 h after birth	423	Peak nSOFA (first 72 h) vs. outcomes
Lewis (2022) [32]	US multicenter	Retrospective	Preterm with NEC (≥IIA)	259	Evaluation; death and surgery/death
Hao (2024) [33]	China	Retrospective	≤32 wk; compare nSOFA vs. CRIB-II vs. SNAPPE-II	759	Admission; mortality; and short-term morbidities

Abbreviations: NICU, neonatal intensive care unit; LOI, late-onset infection; LONS, late-onset neonatal sepsis; LOS, late-onset sepsis; EOS, early-onset infection/sepsis; VLBW, very low birth weight; RDS, respiratory distress syndrome; NEC, necrotizing enterocolitis; T0, time of evaluation; NR, not reported.

**Table 2:** nSOFA performance.

First Author (Year)	Timing Assessed	AUROC/AUC (95% CI)	Reported Threshold/Operating Points or Key Performance Note
Wynn (2020) [24]	T0; +6; +12 h	T0 0.77 (0.62–0.92); +6 0.79; +12 0.93 (0.86–0.997)	≥4 associated with much higher mortality at each window (e.g., +12 h 71% vs. 7%).
Fleiss (2021) [18]	Eval; +6; +12 h	Max(T0/T6) 0.88 (0.84–0.91); center ranges T0 0.71–0.95, T6 0.77–0.96, T12 0.78–0.96	Discrimination improved at +6 versus T0 across centers.
Poggi (2023) [25]	T0; +6; +12; +24 h	At T0, nSOFA 0.950 vs. SIRS 0.569 (*p* = 0.0002)	Best cut-off at T0 = 4; T0 and +6 were independent predictors.
Al Gharaibeh (2025) [26]	T0; +6 h	T0 0.76 (0.71–0.81); +6 0.82 (0.78–0.87)	Cut-off ≥ 2: Se 87%, Sp 66%, NPV 99% (all LOI evals).
Zeigler (2023) [22]	+12 h	0.91 (+12 h)	Compared with HeRO analysis in VLBW infants.
Yeo (2023) * [28]	T0; +6 h; T0–6 max	T0 0.76; +6 0.89; T0–6 max 0.87	EOS cohort; +6 h outperformed T0.
Lewis (2022) * [32]	Eval/serial (NEC)	Death 0.87; Surgery/death 0.84	Discrimination for death and for surgery/death composite.
Hao (2024) [33]	Admission	nSOFA 0.90 vs. SNAPPE-II 0.82 vs. CRIB-II 0.79	nSOFA significantly higher than CRIB-II/SNAPPE-II.
Kurul (2024) [21]	T0; +6; +12 h	NR	At +6 h, aOR per 1-point = 1.31 for 10-day mortality; also associations with BPD/ROP
Wynn (2021) [23]	Admission/serial	“Good-to-excellent” across centers/BW/time	Multicenter validation across 20,152 NICU admissions.
Kraja (2024) [27]	Multiple (−6 to +48 h)	NR	nSOFA > 4 at assessment associated with ~7–16× mortality risk (adj. ~9–18×).
Lobo (2022) [29]	Early after LONS onset	0.92	Brazilian VLBW LONS cohort; strong performance reported.
Berka (2022) * [31]	≤72 h after birth	NR	Early nSOFA predicted mortality/serious morbidity in very preterms.
Shi (2022) * [20]	NICU stay (RDS)	NR	Mortality risk increased per point
Poggi (2025) * [30]	T0; +6; +12; +24 h	NR	Study focused on respiratory outcomes
Xu (2023) * [19]	≤72 h after birth	NR	Study focused on BPD prediction.
Lavilla (2022) * [12]	Hourly kinetics	NR	Organ dysfunction trajectories in extremely preterm infants.

* Non-mortality primary analyses included; Abbreviations: NR, not reported; AUROC/AUC, area under the receiver operating characteristic curve; Se, sensitivity; Sp, specificity; NPV, negative predictive value; SIRS, systemic inflammatory response syndrome; CRIB-II, Clinical Risk Index for Babies II; SNAPPE-II, Score for Neonatal Acute Physiology with Perinatal Extension II; T0, time of evaluation.

**Table 3:** Study-level outcomes.

First Author (Year)	Primary Outcome(s)	Adjusted Per-Point Effect (OR/HR, 95% CI)	Other Non-Overlapping Findings
Wynn (2020) [24]	Mortality (LOS VLBW)	NR	Marked risk separation at ≥4 across T0/+6/+12; +12 h performed best with large mortality gap (71% vs. 7%).
Fleiss (2021) [18]	Mortality (LOI)	NR	Improvement from T0 → +6 observed across centers; center-specific performance broad but consistently “good.”
Poggi (2023) [25]	LONS mortality vs. SIRS	NR	nSOFA at T0 and +6 independently predicted death in multivariate models, outperforming SIRS at T0.
Al Gharaibeh (2025) [26]	LOI-specific mortality	NR	Cut-off ≥ 2 yielded high NPV (99%); effect persisted in VLBW subgroup.
Zeigler (2023) [22]	Sepsis/mortality prediction	NR	Joint analysis with HeRO monitoring; nSOFA retained strong discrimination at +12 h.
Yeo (2023) [28]	EOS mortality	NR	+6 h outperformed T0; early serial assessment was key (T0–6 max better than T0).
Lewis (2022) [32]	NEC death; surgery/death	NR	nSOFA discriminated both death and surgery/death; supports use beyond bacteremia.
Hao (2024) [33]	Mortality ≤ 32 wk	NR	nSOFA outperformed CRIB-II/SNAPPE-II in the same cohort at admission.
Kurul (2024) [21]	10-day mortality; BPD; ROP	aOR 1.31 per point at +6 h (1.22–1.40) mortality; BPD aOR 1.30 (1.13–1.50); ROP aOR 1.24 (1.09–1.41) for nSOFA burden	Burden metric (count of timepoints with nSOFA ≥ 4 from −6 to +48 h) tracked severe morbidity risk.
Shi (2022) [20]	RDS mortality (NICU)	aHR 1.48 per point (1.32–1.67); high vs. low group aHR 19.35 (4.41–84.95)	Mortality rose steeply with increasing nSOFA; results robust after PS-matching.
Xu (2023) [19]	BPD (≤72 h post-birth)	NR (independent association)	Early nSOFA associated with later BPD; broadened use beyond infection episodes.
Poggi (2025) [30]	Respiratory outcomes (LONS)	OR 1.68 (1.34–2.10) for increased respiratory support at +24 h; OR 1.56 (1.21–2.01) for invasive ventilation; OR 1.39 (1.10–1.77) for failure to return to baseline support (all per-point)	nSOFA also associated with severe ROP (OR 1.30, 1.02–1.66).
Berka (2022) [31]	Mortality; serious morbidity (≤72 h)	NR	Early nSOFA within 72 h after birth predicted adverse outcomes in very preterms.
Lobo (2022) [29]	LONS mortality (VLBW)	NR	Strong association between early post-onset nSOFA and sepsis-attributable death in Brazilian cohort.
Kraja (2024) [27]	LOS mortality (very preterm)	NR	Scores > 4 linked to ~7–16× crude and ~9–18× adjusted mortality odds across windows.
Wynn (2021) [23]	All-cause NICU mortality	NR	Good–excellent discrimination across BW strata and time after admission in 20k+ infants.
Lavilla (2022) [12]	Adverse outcomes in extremely preterm	NR	Hourly organ dysfunction trajectories (nSOFA components) linked with adverse outcomes; supports serial monitoring.

Abbreviations: NR, not reported; OR, odds ratio; aOR, adjusted odds ratio; HR, hazard ratio; aHR, adjusted hazard ratio; BPD, bronchopulmonary dysplasia; ROP, retinopathy of prematurity; LONS, late-onset neonatal sepsis; LOI, late-onset infection; LOS, late-onset sepsis; EOS, early-onset infection/sepsis; NICU, neonatal intensive care unit.

## Data Availability

The original contributions presented in this study are included in the article/Supplementary Materials. Further inquiries can be directed to the corresponding authors.

## Supplementary Materials

The following supporting information can be downloaded at: https://www.mdpi.com/article/10.3390/diagnostics16020349/s1.
